# Sanitary measures in piggeries, awareness, and risk factors of African swine fever in Benue State, Nigeria

**DOI:** 10.1007/s11250-018-1764-7

**Published:** 2018-12-19

**Authors:** A. Asambe, A. K. B. Sackey, L. B. Tekdek

**Affiliations:** 1 Department of Animal Science, Faculty of Agriculture and Agricultural Technology, Federal University Dutsina, Dutsina, Katsina State P.M.B 5001 Nigeria; 2 Department of Veterinary Medicine, Faculty of Veterinary Medicine, Ahmadu Bello University, Zaria, Nigeria

**Keywords:** African swine fever, Awareness, Piggery, Risk factors, Sanitary measures

## Abstract

The present study describes assessment of sanitary measures in piggeries of Benue State, Nigeria, to identify the risk factors of African swine fever. Questionnaires were distributed to 74 respondents consisting of piggery owners and attendants in different piggeries across 12 local government areas (LGAs) to collect data for this study. Sanitary measures in piggeries were observed to be generally very poor, though respondents admitted being aware of ASF. Piggeries located within 1-km radius of a slaughter slab (OR = 9.2, 95% CI 3.0–28.8; *p* < 0.0001) and piggeries near refuse dump sites (OR = 3.0, 95% CI 1.0–9.5; *p* < 0.05) showed higher chances of African swine fever virus (ASFV) infection, while piggeries where farm workers wear their work clothes outside of the piggery premises (OR = 0.2, 95% CI 0.1–0.7; *p* < 0.01) indicate less chances of infection but had a significantly associated *p* value thus were identified as potential risk factors. The study concluded that pigs in Benue State are still at risk of an ASF outbreak. Proper sanitary and hygienic practices are advocated and emphasized in piggeries, while routine surveillance for African swine fever virus antibodies in pigs in Benue State is strongly recommended to provide a reliable reference database to plan for the prevention of any devastating ASF outbreak.

## Introduction

African swine fever (ASF) is an infectious disease that affects both domestic and wild pigs (Montgomery 1921; OIE 2005). The hemorrhagic and transboundary disease is caused by African swine fever virus (ASFV), belonging to the genus *Asfivirus* and currently the only member of the family *Asfarviridae* (Dixon et al. 2000). Both domestic and wild pigs, as well as soft tick of the genus *Ornithodoros* are reservoirs of the ASF virus (Plowright et al. 1969; Thomson et al. 1981).

African swine fever control is difficult probably due to lack of portent vaccine, the inability of the infection to elicit the production of neutralizing antibodies, and wider coverage in terms of distribution of the soft-tick vector *Ornithodoros* species (Boshoff 2003). ASF is reported to have had severe socioeconomic impact in endemic areas as well as on naïve pigs especially where it is newly introduced threatening food security both at household and commercial levels with the consequent mortality and trade restrictions (Lubisi 2005; Etters et al. 2011).

The endemicity of ASF in Nigeria (Etters et al. 2011) and indeed Benue State (Asambe et al. 2017; El-Hicheri 1998), where intermittent infections are experienced has wiped out pig herds (OIE, 2005) over the years. ASF has remained a problem in Nigerian piggeries since 1997 (El-Hicheri 1998; FAO 1998; Babalobi et al. 2003; Babalobi et al. 2007) where persistent infections with ASFV appear to recur in core pig-producing areas of the country (Fasina et al. 2012), thereby adversely affecting the bustling and rising activities in the industry (Babalobi et al. 2007).

The continued presence and maintenance of the virus in domestic pig populations pose an enormous problem thus prompting a cause for greater understanding of the factors responsible. Hence, the need to identify the risk factors, inappropriate practices, and sanitary measures in piggeries that contribute to the spread and maintenance of ASF as these are vital for achieving control and eradication.

## Materials and methods

### Study area

Benue state is located in the north central region of Nigeria, a farming zone known for high pig production principally raised by small holder pig farmers operating semi-intensive pig production system. The state has a tropical sub-humid climate, with two distinct seasons namely the wet and dry seasons, with annual rainfall within the range of 100–200 mm. Temperatures are generally very high during the day, particularly in March and April (BNSG, 2011). Sampling locations include: Apa, Gboko, Gwer-west, Katsina Ala, Kwande, Makurdi, Obi, Ogbadibo, Oturkpo, Tarka, Ukum, and Vandeikya local government areas (LGAs). A total of 74 piggeries were sampled for the study.

## Questionnaire design and administration

A pre-tested structured, interviewer-administered questionnaire was used to obtain data on sanitary measures in piggeries, awareness, and risk factors of ASF. A respondent was someone who was actively involved in the daily activities of the piggery not necessarily the piggery owner.

## Data analysis

The results obtained were analyzed by the Statistical Package for Social Sciences (SPSS) version 20.0. Descriptive statistics and univariate analysis (chi-square and odds ratios) were used to test for association between categorical variables. *p* values ≤ 0.05 were considered significant.

## Results and discussion

All the 74 piggeries had quarantine or an isolation unit within 100-m radius of the regular pigpen, whereas fewer respondents complied with some of the basic biosecurity and sanitary measures necessary for the control of ASF as outlined in Table 1. It was observed that respondents generally had very poor sanitary and biosecurity measures towards containment and prevention of ASF infection. The observed differences in the level of compliance with the assessed biosecurity and sanitary measures between seropositive and seronegative piggeries in this study is in agreement with previous report by Awosanya et al. (2015) who had used similar indices of measurement in their study.

**Table 1:** Level of compliance with sanitary measures in piggeries

Measures	Yes (%)
Quarantine section within 100 m of the main property	74 (100)
Designated work clothes for the piggery	17 (23.0)
Workers bath in the piggery after work	27 (36.5)
Lend out service boars	62 (83.8)
Clean (wash/sweep) pen floor daily	12 (16.2)
Disinfect pen floor daily	12 (16.2)
Clean (wash) work utensils daily	74 (100)
Carcass burial within 1-km radius	74 (100)
Piggery designated footwear	12 (16.2)
Routine pests control	12 (16.2)
Access by stray animals	28 (37.8)
Presence of rodents on the piggery	74 (100)

Table 2 summarizes results of respondents’ level of awareness of ASF and its associated signs expressed in percentages for the 74 respondents interviewed whereas all (100%) had admitted being aware of ASF. This is indicative of the respondents’ admittance to high level of awareness of ASF with the resultant poor practices towards the disease control. Although ASF is recognized as a major limiting factor for pig production in sub-Saharan Africa, Adesehinwa et al. (2003), Mashatise et al. (2005), Halimani et al. (2007), Ironkwe and Amefule (2008), Kagira et al. (2010), Karimuribo et al. (2011), Moreki and Mphinyane (2011), Nwanta et al. (2011), Petrus et al. (2011), and Muhanguzi et al. (2012) have suggested or identified lack of adequate knowledge and information about pig production and health as the most serious constraints to pig production and improved management practices in piggeries, which is in agreement with our present study.

**Table 2:** Awareness of ASF admitted by respondents

Category	Number of respondents	Yes (%)
Have you ever heard of ASF?	74	74 (100)
Common Signs of ASF Aware of by Respondents		
Abortion	74	10 (13.5)
Hyperemia	74	51 (68.9)
Weakness	74	13 (17.6)

The most common ASF-related signs mentioned by the respondents were hyperemia (reddening of skin) 51 (68.9%) followed by weakness or unwillingness of the pigs to stand 13 (17.6%) and abortion 10 (13.5%) respectively (Table 2). These associated signs as mentioned by the respondents may or may not be due to ASF but has shown the ability of the farmers to recognize such associated signs and therefore assist in early detection of an ASF infection. However, this may be impeded by the farmers’ unwillingness to report outbreaks which possibly could be explained by the adjudged low compensation by the government, if any, for the culling of affected pigs but it should be noted that early detection and reporting is critical to ASF control and eradication (Sánchez-Vizcaíno 2010) and hence the farmers should be made aware of this.

Table 3 summarizes the results of the risk factors associated with ASF infection in the sampled piggeries across Benue State expressed in chi-square and odds ratios with the associated confidence intervals. The results show that location of piggery within 1-km radius of a slaughter slabs had 9.2 (95% CI 3.0–28.8) more chances of getting infected and was significantly associated (*p* < 0.0001) (Table 3). Location of piggeries within 1-km radius of pig slaughter slab was found to be statistically significant and identified as a risk factor. This may be as a result of pig farmers presenting sick and unthrifty pigs for slaughter at abattoirs first without determining the cause of sickness, to which some may be ASF (Randriamparany et al. 2005; Fasina et al. 2010) thus contributing to ASF spread in nearby piggeries (Costard et al. 2009). Since ASF virus is present in tissues and body fluids of slaughtered sick pigs, massive environmental contamination, and possible nearby piggery infection may result. Similarly, rodents and wild birds are usually observed around open slaughter slabs environment and they carry away intestinal content and viscera, which some are infectious and are disposed of indiscriminately to nearby piggeries thus facilitating the infection of naïve pigs. Also, farmers often participate in various processes on slaughter slab floors with the consequent risk of carrying the virus to their piggeries with resultant infection as reported by Fasina et al. (2012) in major pig producing areas in Nigeria.

**Table 3:** Risk factors associated with African swine fever

Category	OR (95% CI)	χ^2^/p values
Slaughter slab within 1-km radius of the pig farm	9.2 (3.0–28.8)	(χ^2^ = 20.704, p = 0.000)
Refuse dump sites within 1 km radius of the pig farm	3.1 (1.0–9.5)	(χ^2^ = 4.458, p = 0.035)
Wearing of work clothes outside of the piggery premises	0.2 (0.1–0.7)	(χ^2^ = 7.179, p = 0.007)
Sharing of farm workers with other pig farms	Constant	Constant
Sharing of working utensils with other pig farms	Constant	Constant
Source of replacement stock	1.3 (0.3–6.1)	(χ^2^ = 0.121, p = 0.728)
Feeding of swill to pigs	0.5 (0.1–2.2)	(χ^2^ = 0.910, p = 0.340)
Nearby pig farm within 1 km radius of each other	1.3 (0.3–6.1)	(χ^2^ = 0.135, p = 0.714)
Presence of functional foot dip on the pig farm	1.0 (0.1–7.5)	(χ^2^ = 0.002, p = 0.962)
Presence of ticks on pigs	0.7 (0.2–3.2)	(χ^2^ = 0.210, p = 0.647)
Pig farm perimeter fencing	0.7 (0.1–5.6)	(χ^2^ = 0.108, p = 0.743)

Pig farms located within 1-km radius of refuse dump sites were identified as risk factors with statistically significant association (*p* < 0.05) and had 3.0 (95% CI 1.0–9.5) more chances of getting infected (Table 3). This result is in agreement with the findings of (Awosanya et al. 2015) and may be related directly to a local spread between and within piggeries and may occur through direct pig-to-pig contact, or by fomites especially in scavenging populations and possibly by stray animals such as dogs and pigs gaining access to the nearby piggeries (Fasina et al. 2012).

Also farms where farm workers were reported to wear their work clothes outside of the piggery was significantly associated with ASF infection (*p* < 0.01) with 0.2 (95% CI 0.1–0.7) though with an odds ratio indicating less chances of getting infected (Table 3). This results was similarly observed by (Awosanya et al. 2015) and could be explained by the fact that ASF is reported to be transmitted by indirect contact through fomites though this mode of transmission is said to be efficient in a very high viral load (Mur et al. 2012; de Carvalho et al. 2013). The movement of work clothes and contacts in and out of the farm at regular interval is suspected to serve as a vehicle for transmission.

The overall implication of the research findings is that these may further create a conducive environment for some vectors and subsequently increase the spread of the African swine fever virus. Individuals, corporate organizations, and governmental agencies must collaborate to create awareness on the need in adhering to standard practices and possibly provide grant to piggery farmers so as to curtail the devastating effect of ASF outbreaks.

## Conclusion

The study concluded by identifying ASF risk factors in the study area as presence of slaughter slab within 1-km radius to the piggery, presence of refuse dump sites within 1-km radius to piggery, and wearing of designated work clothes outside the piggery premises and also suggest that pigs in Benue State are still at risk of an ASF outbreak. Strict adherence to hygienic and proper sanitary measures in piggeries, routine surveillance, and monitoring of ASFV antibodies in pigs in Benue State to provide a comprehensive and readily accessible database to plan for the prevention of any fulminating outbreak is therefore recommended.
